# hFcγRIIa: a double-edged sword in osteoclastogenesis and bone balance in transgenic mice

**DOI:** 10.3389/fimmu.2024.1425670

**Published:** 2024-08-30

**Authors:** Jie Miao, Hong-Min Wang, Xiao-Hua Pan, Zheng Gong, Xiao-Ming Gao, Fang-Yuan Gong

**Affiliations:** School of Biology and Basic Medical Sciences, Suzhou Medical College, Soochow University, Suzhou, China

**Keywords:** FcγRs, hFcγRIIa, osteoclastogenesis, rheumatoid arthritis, IV.3

## Abstract

Rheumatoid arthritis (RA) is a chronic autoimmune disease accompanied by local and systemic bone loss. FcγRs, especially FcγRIIa (hFcγRIIa), have been implicated in the pathogenesis of RA. However, the contribution of hFcγRIIa to bone loss has not been fully elucidated. In the present study, we demonstrated the double-edged sword role of hFcγRIIa on osteoclast differentiation through investigations involving hFcγRIIa-transgenic (hFcγRIIa-Tg) mice. Our findings reveal that hFcγRIIa-Tg mice, previously shown to exhibit heightened susceptibility to collagen-induced arthritis (CIA), displayed increased osteoporosis during CIA or at advanced ages (40 weeks), accompanied by heightened *in vivo* osteoclast differentiation. Notably, bone marrow cells from hFcγRIIa-Tg mice exhibited enhanced efficiency in differentiating into osteoclasts and bone resorption *in vitro* compared to wild-type mice when stimulated with receptor activators of NF-κB ligand (RANKL). Additionally, hFcγRIIa-Tg mice exhibited augmented sensitivity to RANKL-induced bone loss *in vivo*, highlighting the osteoclast-promoting role of hFcγRIIa. Mechanistically, bone marrow cells from hFcγRIIa-Tg mice displayed heightened Syk self-activation, leading to mTOR-pS6 pathway activation, thereby promoting RANKL-driven osteoclast differentiation. Intriguingly, while hFcγRIIa crosslinking hindered RANKL-induced osteoclast differentiation, it activated the kinase cAbl, subsequently triggering STAT5 activation and inhibiting the expression of osteoclast-associated genes. This study provides novel insights into hFcγRIIa-mediated osteoclast biology, suggesting promising therapeutic targets for managing bone remodeling disorders.

## 1 Introduction

Rheumatoid arthritis (RA) is an autoimmune disease characterized by chronic joint inflammation and local bone erosion (1, 2). Notably, RA patients commonly experience systemic bone loss, even in the disease’s early stages (3). While genetic and environmental factors contribute to RA’s etiology, the precise mechanisms underlying bone destruction, a hallmark of this condition, remain incompletely understood (4–6). Osteoclasts are specialized bone resorbing cells that control both generalized and focal bone loss in RA patients. Beyond their role in bone resorption, osteoclasts actively participate in inflammatory and autoimmune responses, with infiltrating immune cells (T cells, B cells, neutrophils, and macrophages) notably influencing synovial inflammation (1, 3, 7). The presence of autoantibodies, notably rheumatoid factor (RF) and anti-citrullinated protein antibodies (ACPA), not only aids in RA diagnosis but also links to disease pathogenesis, particularly through the Fc region of these autoantibodies (3, 8).

Autoantibodies, forming immune complexes (ICs) with self-antigens, accelerate bone loss via Fcγ receptors (FcγRs) present on osteoclast precursor cells (OCPs) (9–12). FcγRs are cell surface glycoproteins that sense the Fc region of IgG antibodies. In mice, there are four different classes of FcγRs (FcγRI, FcγRIIb, FcγRIII, and FcγRIV), while in humans, six different classes have been identified (FcγRI, FcγRIIa, FcγRIIb, FcγRIIc, FcγRIIIa, and FcγRIIIb). FcγRIIb is the sole inhibitory FcγR known to suppress cellular activation via an immunoreceptor tyrosine-based inhibition motif (ITIM) (13). Investigating the role of FcγRs in osteoclastogenesis and bone resorption in inflammatory disorders is challenging and influenced by multiple factors, including the degree of IgG aggregation and affinity for each FcγR (14, 15), the direct or indirect effect of ICs, the inflammatory microenvironment and the species. Several studies have dissected the roles of individual FcγRs and demonstrated that they may play dual roles in osteoclastogenesis under various conditions. Activating FcγRI and FcγRIV in mice has no effect on bone homeostasis or OC differentiation under physiological conditions (9, 16). However, in the inflammatory arthritic microenvironment, FcγRI and FcγRIV stimulation enhances OC differentiation and activity both *in vitro* and *in vivo* (9, 17). FcγRIIb^–/–^ mice developed osteoporosis, but only in the presence of hypergammaglobulinemia or after systemic injection of IgG ICs. Similarly, FcγRIII promotes osteoclastogenesis under inflammatory conditions (9).

Most activating FcγRs that activate intracellular signaling involve a ligand-binding α chain and an FcRγ chain with an immunoreceptor tyrosine-based activation motif (ITAM) (18, 19). It is widely assumed that activating FcRγ chain promotes RANKL-induced osteoclastogenesis and enhances bone destruction (17). FcRγ and DAP12, which harbor ITAM-bearing adaptor proteins, have also been reported to play crucial roles in the transduction of costimulatory signals from RANK, thus promoting RANKL-mediated osteoclast differentiation (9).

Among these active receptors, hFcγRIIa emerges uniquely, operating without the common FcRγ chain and containing its ITAM signaling in the ligand-binding chain (20). It has been reported that hFcγRIIa, particularly hFcγRIIa-R131, is associated with the development of RA in humans (21). Animal studies have indicated that hFcγRIIa is associated with spontaneous autoimmune inflammation and plays a crucial role in joint bone erosion (22). However, whether hFcγRIIa can directly regulate OCs has not been thoroughly investigated. In a previous study, we demonstrated that plate-bound IgG could induce nonclassical OC differentiation in human monocytes through hFcγRIIa (23). In this study, we further discuss the role of hFcγRIIa in OC differentiation and its contribution to bone loss using an hFcγRIIa transgenic mouse model.

## 2 Materials and Methods

### 2.1 Mice

Human FcγRIIa transgenic (hFcγRIIa-Tg) mice [B6; SJL-Tg (FCGR2A)11Mkz/J] and wild-type (WT) mice [B6; SJL] were purchased from The Jackson Laboratory. The heterozygous hFcγRIIa-Tg (hFcγRIIa^+/−^) female mice used in this study were generated from transgenic and wild-type (WT) offspring and genotyped (modified The Jackson Laboratory protocol). Mice were housed under specific pathogen-free (SPF) conditions. All animal experiments were approved by the Ethics Committee of Soochow University and were performed following the guidelines of the Institutional Animal Care and Use Committee of Soochow University.

### 2.2 Microcomputed tomography analysis

Mouse femur bones were collected and fixed in 4% paraformaldehyde overnight, followed by storage in 70% ethanol before μCT analysis. µCT images of femurs were scanned by using a high-resolution µCT system Skyscan 1176 (Bruker microCT, Belgium) with a resolution of 9 μm (source voltage: 50 kV, source current: 500 µA, rotation step: 0.7 deg) at the Institute of Orthopaedics at Soochow University. Raw images between 540 μm and 1890 μm (150 slices) away from the epiphyseal plate of femurs were manually designated as regions of interest (ROIs) and reconstructed into three-dimensional reconstructions using the reconstitution software CT Analyser (CTAn) to assess trabecular bone morphology. The morphometric parameters included the bone mineral density (BMD), bone volume/tissue volume (BV/TV), trabecular bone pattern factor (Tb.pf), trabecular thickness (Tb.Th), trabecular number (Tb.N), and trabecular separation (Tb.Sp).

### 2.3 Bone histomorphometric analysis

Femur bones were dissected and fixed in 4% paraformaldehyde overnight and decalcified in 10% EDTA buffer for 3 to 4 weeks until the bone was flexible. Serial paraffin sectioning (4 μm) and TRAP staining were performed by Wuhan Servicebio Technology Co., Ltd. Digital images were taken with an Eclipse E100 microscope (Nikon, Japan) at 40× magnification, and the osteoclast surface/bone surface (OS/BS) were quantified using ImageJ.

### 2.4 Osteoclast differentiation

Bone marrow cells (BMCs) were isolated from the femurs and tibias of 6- to 8-week-old female mice by flushing the bones with PBS followed by treatment with red blood cell lysis buffer (Beyotime, China). BMCs were cultured on 10 cm petri dishes (Nunc, USA) overnight in α-MEM (Corning, USA) supplemented with 10% FBS (Biological Industries, Israel) and a low concentration of 10 ng/mL M-CSF (PeproTech, USA) in an incubator with 5% CO_2_ at 37°C. The next day, the non-adherent cells were resuspended and cultured in 96-well plates (Corning, USA) at a density of 8×10^4^ cells/200 μL/well with 50 ng/mL M-CSF for 3 days to generate bone marrow macrophages (BMMs). After washing three times with PBS to remove non-adherent cells, BMMs were further induced to generate OCs with 50 ng/mL M-CSF and 50 ng/mL RANKL (PeproTech, USA) for 3 to 5 days, and the medium was changed every other day. This type of differentiation is referred to as the conventional culture system. In the direct differentiation of bone marrow into OCs experiments, the non-adherent cells as described above were resuspended and cultured in 96-well plates at a density of 8×10^4^ cells/200 μL/well with 50 ng/mL M-CSF and 50 ng/mL RANKL for 3 to 5 days to generate OCs. In some other experiments, BMCs were cultured on plate-coated anti-human CD32 antibody, clone IV.3 (Stem Cell, Canada) or mouse IgG2b (BioLegend, USA) in the presence or absence of some inhibitors (Selleck, USA) during differentiation. These inhibitors include a Syk inhibitor (R406), an mTOR inhibitor (rapamycin), a Src/Abl inhibitor (bosutinib, BOS), and a STAT5 inhibitor (STAT5i).

### 2.5 Tartrate-resistant acid phosphatase staining

After differentiation, the cells were washed with PBS and fixed with 4% formaldehyde containing acetone and citrate solution for 3 minutes at room temperature. After aspirating the fixative solution, the cells were washed three times with deionized water and stained for TRAP by using a histochemical kit (Sigma-Aldrich, USA) according to the manufacturer’s instructions. In addition, the nuclei were counterstained with DAPI (Roche, Switzerland). Raw images were captured by using an inverted phase-contrast microscope (Nikon, Japan) at 10× or 40× magnification in three randomly chosen fields of view per well. TRAP-positive and multinucleated (≥3 nuclei) cells (MNCs) were identified as OCs and quantified by using ImageJ software.

### 2.6 Immunofluorescence staining

To assess the formation of F-actin rings, OCs were washed twice with PBS and fixed with 4% paraformaldehyde for 10 minutes at room temperature. After washing three times with PBS, the fixed cells were incubated with PBS containing 0.5% Triton X-100 (Amresco, USA) for 5 minutes at room temperature. After washing three times with PBS, the cells were treated with 1% BSA containing TRITC-phalloidin (Yeasen, China) for 30 minutes with light protection. In addition, cell nuclei were counterstained with DAPI (Roche, Switzerland). F-actin rings were visualized by using a fluorescence microscope (Nikon Ti, Japan).

### 2.7 Bone resorption assay

To assess osteoclast-mediated bone resorption *in vitro*, BMCs were cultured on 96-well Osteo Assay Stripwell plates (Corning, USA) coated with a proprietary hydroxyapatite mineral surface at 1×10^5^ cells/200 μL/well and treated with 50 ng/mL M-CSF in the presence or absence of 50 ng/mL RANKL for 15 days, after which the α-MEM was changed every other day. Von Kossa staining was performed to detect bone resorption pits. After aspirating the medium, deionized water was added to the wells, after which the cells were lysed for 2 hours. The plate wells were immersed in 5% silver nitrate solution under UV light for 30 to 60 minutes until a dark color emerged. The silver nitrate solution was removed, the mixture was washed three times with deionized water, and 5% thiosulfate sodium solution was added to neutralize the remaining nitrate solution. After drying, the resorption pits were imaged by using inverted phase-contrast microscopy (Nikon Ti, Japan) and quantified using ImageJ software.

### 2.8 Collagen-induced arthritis model

WT and hFcγRIIa-Tg mice (8 weeks old, female) were immunized intradermally at the base of the tail with bovine type II collagen (CII) (Chondrex, USA) emulsified in complete Freund’s adjuvant (Difco, USA). Three weeks after the primary immunization, the mice were booster injected with the CII/complete Freund’s adjuvant (Difco, USA) emulsion. Mice were examined for up to 45 days, and arthritis was scored using the following criteria: 0, no joint erythema or swelling; 1, erythema or mild swelling restricted to the tarsal or ankle joint; 2, erythema and mild swelling extending from the ankle to tarsals; 3, erythema and mild swelling extending from the ankle to the metatarsal; and 4, erythema and severe swelling extending from the ankle, foot, and fingers. The scores for all the fingers of the forepaws and hind paws, wrists, and ankles were totaled for each mouse, with a maximum possible score of 16 for each.

### 2.9 sRANKL-induced bone loss mouse model

To assess osteoclast-induced bone loss in hFcγRIIa-Tg mice, we used a soluble RANKL (sRANKL)-induced rapid bone loss mouse model (24). A total of twenty female mice (8 weeks old) were randomly assigned to four groups (n=5): the WT vehicle group, the Tg vehicle group, the WT sRANKL group, and the Tg sRANKL group. In brief, the mice were injected intraperitoneally with sRANKL (2.0 mg/kg) or an equal volume of PBS at 24-hour intervals for 3 days. All mice were sacrificed 1.5 hours after the final injection, and serum samples and femur bones were harvested for analysis.

### 2.10 Enzyme-linked immunosorbent assay (ELISA)

Mouse blood samples were collected and kept at room temperature for 30 to 60 minutes to allow clotting before centrifugation at 2000 × g for 10 minutes at 4°C and then stored at -80°C until use. The serum concentration of tartrate-resistant acid phosphatase 5b (TRAP-5b) was measured by an ELISA kit (Kejing, China) according to the manufacturer’s instructions.

### 2.11 Flow cytometry

BMCs were isolated from the femurs and tibias of 6- to 8-week-old female mice by flushing the bones with PBS, followed by hypotonic red cell lysis using red blood cell lysis buffer (Beyotime, China). After blocking, the cells were incubated with a mixture of fluorescence-labelled antibodies for 30 minutes on ice. PerCp/Cy5.5-conjugated anti-mouse Ter119, PerCp/Cy5.5-conjugated anti-mouse CD3, PerCp/Cy5.5-conjugated anti-mouse B220, APC/Cy7-conjugated anti-mouse F4/80, FITC-conjugated anti-mouse CD11b, APC-conjugated anti-mouse CD115, PE-conjugated anti-mouse CD265, and APC/Cy7-conjugated anti-mouse Ly6C (BioLegend, USA) were used. The stained cells were washed twice with PBS, resuspended in 300 μL of PBS and subsequently analyzed using an Attune NxT flow cytometer (Life Technologies, USA) and FlowJo software.

### 2.12 Real-time quantitative PCR

Total RNA was extracted (OMEGA, USA) and reverse transcribed (Takara, Japan) following the manufacturer’s protocol. Real-time quantitative PCR was performed with SYBR Green (Takara, Japan) on a StepOne Plus real-time PCR system (Applied Biosystems, USA). The level of mRNA expression was calculated by the 2^-△△Ct^ method and normalized to that of GAPDH. The sequences of primers used are listed in **Supplementary Table S1**.

### 2.13 Western blot analysis

At the end of the culture experiments, the cells were washed with PBS and lysed with RIPA buffer (Beyotime, China) supplemented with a protease inhibitor cocktail (Roche, Switzerland). The cell lysates were boiled in the presence of 5× loading buffer for 10 minutes and incubated on ice until use. Proteins were separated by SDS-PAGE (10% to 15% polyacrylamide gels) and then transferred to polyvinylidene difluoride (PVDF) membranes. After blocking with 5% skim milk in PBST for 1 hour, the membranes were probed with primary antibodies against GAPDH, Syk, p-Syk, p-S6, p-c-fos, NFATc1 (Cell Signaling Technology, USA) or NFATc1 (Santa Cruz Biotechnology, USA) overnight at 4°C. After washing with PBST, the membranes were further incubated with an appropriate HRP-labelled goat anti-rabbit or mouse secondary antibody (Southern Biotech, USA) for 1 hour at room temperature, followed by detection with an enhanced chemiluminescence (ECL) plus Western blotting Detection System (Tanon 5200, China).

### 2.14 Statistical analysis

All the data are presented as the mean ± s.e.m. of at least three separate experiments. Statistical analyses were performed with GraphPad Prism 5.0 software using 2-tailed tests, and statistical comparisons were performed using Student’s t test or one-way ANOVA. A value of p < 0.05 was considered to indicate statistical significance. In the figures, * represents p<0.05, ** represents p<0.01, *** represents p<0.001, and n.s represents not significant.

## 3 Results

### 3.1 hFcγRIIa-Tg mice developed osteoporosis in the CIA model

**Figure 1:**
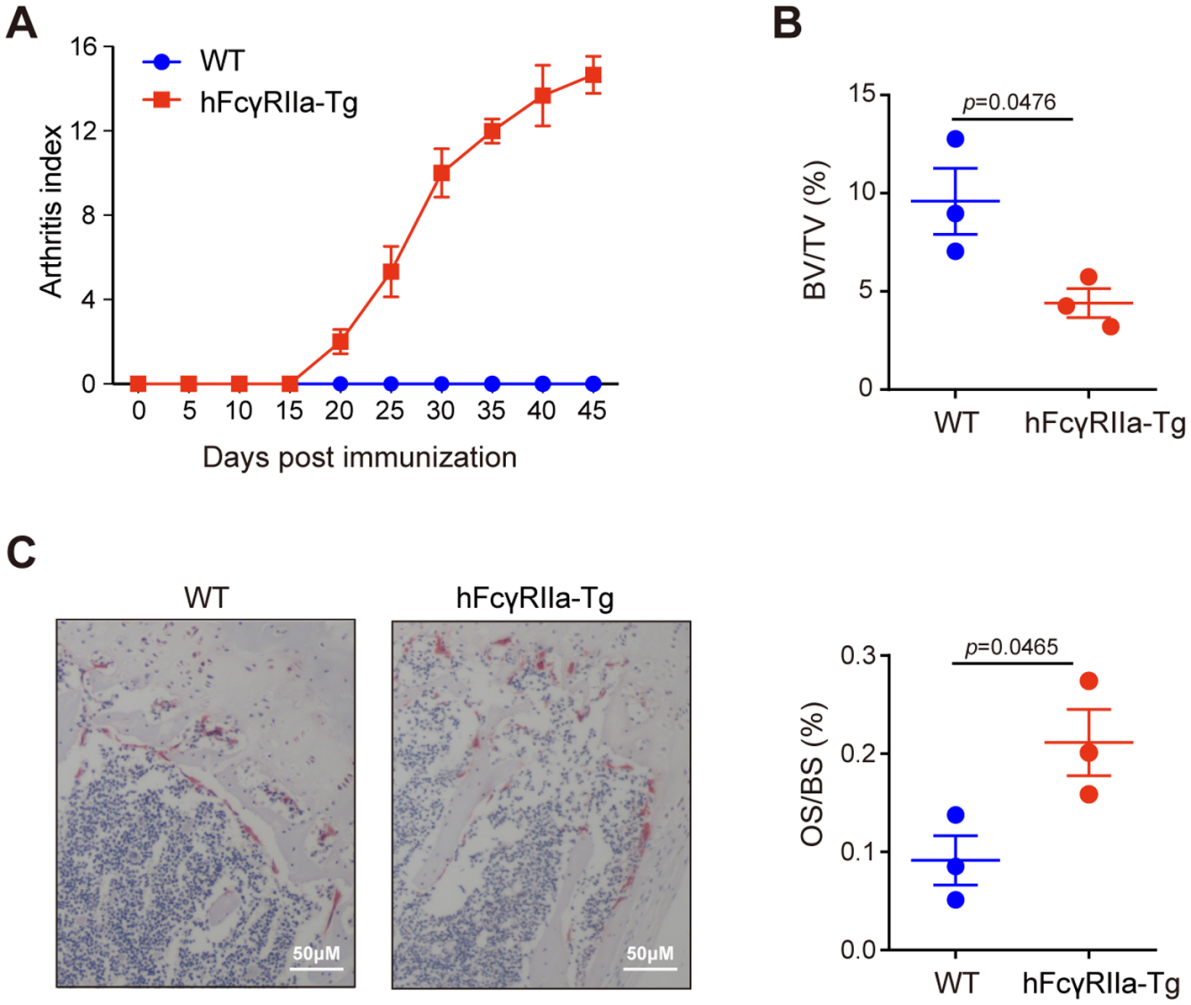
Bone loss in hFcγRIIa-Tg CIA mice. **(A)** CIA in hFcγRIIa-Tg mice. hFcγRIIa-Tg and WT mice were injected twice with CII emulsified in Freund’s adjuvant. The immunized mice were observed daily and scored for the development of arthritis until day 45. **(B)** Micro-CT (μCT) analysis. Femur bones were scanned via μCT. The bone volume/tissue volume (BV/TV) was also analyzed. **(C)** Representative images of TRAP-stained femur bone sections (40× magnification). The osteoclast surface/bone surface (OS/BS) is shown in the right panel. The results are shown as mean ± SD of three pairs of mice.

The development of osteoporosis in hFcγRIIa-Tg mice within the CIA model signifies the pivotal role of hFcγRIIa in disease pathogenesis. Specifically expressed on human myeloid cells, hFcγRIIa was investigated in hFcγRIIa-Tg mice to delineate its contribution to disease pathophysiology *in vivo*. Notably, hFcγRIIa-Tg mice exhibited heightened susceptibility to collagen-induced arthritis (CIA) compared to WT mice (**Figure 1A**), a susceptibility effectively reversed by the blockade of hFcγRIIa (22). In addition to evident bone erosion observed in the ankle joint, micro-CT (μCT) analysis unveiled osteoporosis in the femurs of collagen-immunized hFcγRIIa-Tg mice. Trabecular bone analysis revealed a statistically significant decrease of approximately 50% in trabecular bone volume in hFcγRIIa-Tg mice compared to WT mice (**Figure 1B**). To further assess the impact on osteoclasts in CIA mice, bone histomorphometry and TRAP staining were conducted, revealing a significant increase in the number of osteoclasts (OS/BS) on the trabecular bone surface of hFcγRIIa-Tg mice compared to WT mice (**Figure 1C**). Taken together, these data indicated that hFcγRIIa may contribute to osteoporosis in the CIA model.

### 3.2 hFcγRIIa contribution to osteoporosis in aging mice

**Figure 2:**
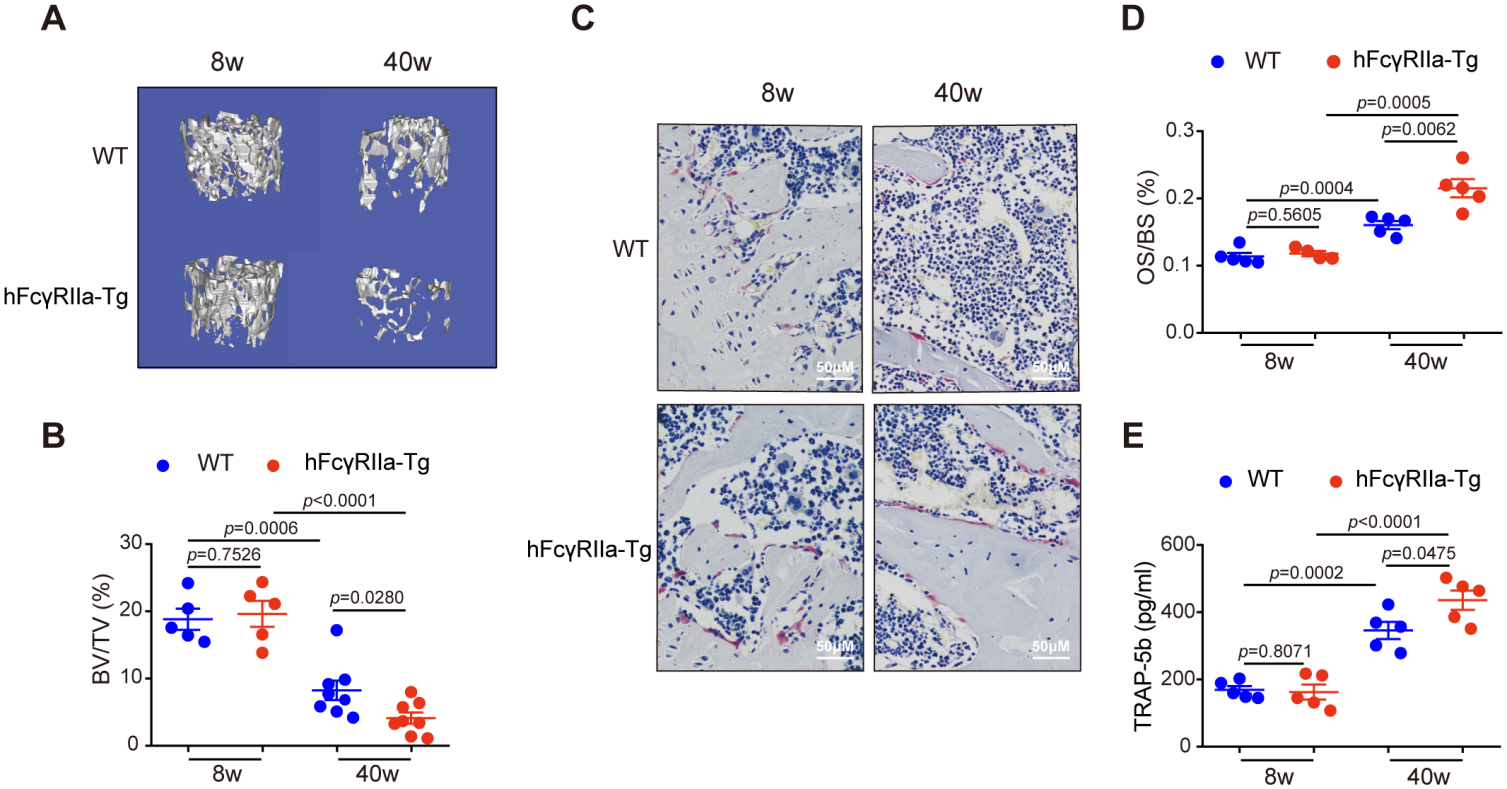
hFcγRIIa-Tg mice exhibit enhanced osteoporosis compared with WT mice. **(A, B)** The femurs of 8- and 40-week-old hFcγRIIa-Tg and WT mice were scanned via μCT. Representative 3D reconstructions of trabecular bone in femurs of 8- and 40-week-old female mice are shown in **(A)** The BV/TV was analyzed in **(B)**. **(C, D)** Representative images of TRAP-stained femur bone sections (40× magnification) in **(C)** OS/BS are shown in **(D)**. **(E)** TRAP-5b in the serum was determined via ELISA. The results are shown as mean ± SD of at least five pairs of mice.

To probe hFcγRIIa’s involvement in osteoporosis, we compared bone metabolism in aging hFcγRIIa-Tg mice to that in WT mice. Notably, μCT imaging of femurs at 40 weeks revealed an osteopenic phenotype in hFcγRIIa-Tg mice, while bone parameters didn’t differ between hFcγRIIa-Tg and WT mice at 8 weeks. At 40 weeks, both hFcγRIIa-Tg and WT mice displayed significantly lower bone mass compared to 8 weeks. However, the decline in bone volume was markedly pronounced in hFcγRIIa-Tg mice at 40 weeks (**Figures 2A, B**; **Supplementary Figures 1A, B**). Furthermore, analysis at 40 weeks unveiled a significantly higher number of osteoclasts per bone surface in hFcγRIIa-Tg mice than in their WT counterparts, indicating the osteopenic phenotype stemmed from an augmented abundance of osteoclasts *in vivo* (**Figures 2C, D**). This observation was corroborated by elevated serum levels of TRAP-5b, a marker of osteoclast number, in hFcγRIIa-Tg mice at 40 weeks (**Figure 2E**). These findings strongly suggest that hFcγRIIa hastens bone resorption by bolstering osteoclastogenesis during the aging process, contributing significantly to the onset of osteoporosis.

### 3.3 OC differentiation was more efficient from macrophages in hFcγRIIa-Tg mice *in vitro*

**Figure 3:**
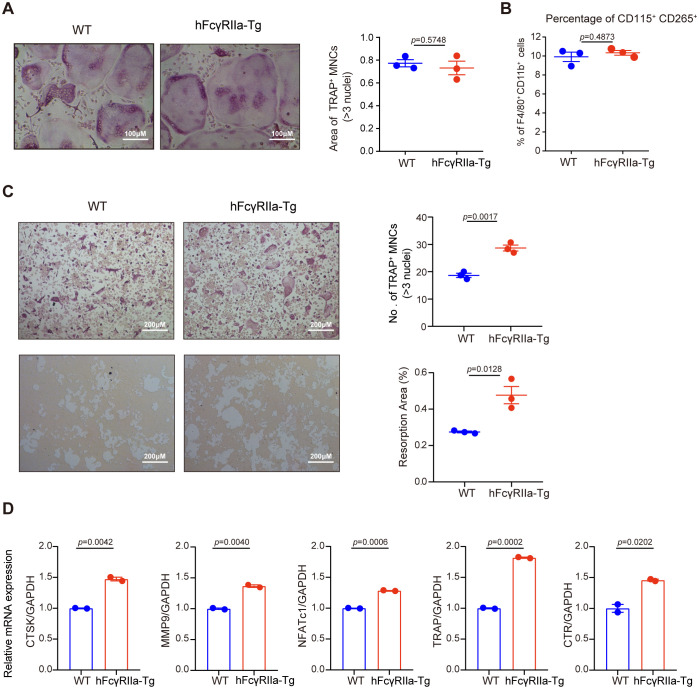
Comparison of the OC differentiation efficiency of BMCs from hFcγRIIa-Tg and WT mice *in vitro*. **(A)** BMCs from WT or hFcγRIIa-Tg mice were simultaneously exposed to M-CSF (50 ng/ml) and RANKL (50 ng/ml). After/span>incubating for 3 days, the osteoclasts were stained for TRAP, and TRAP-positive osteoclasts containing three or more nuclei were counted. **(B)** The proportion of CD115^+^ CD265^+^ in the BMMs of WT or hFcγRIIa-Tg mice. **(C)** BMCs from WT or hFcγRIIa-Tg mice were cultured with M-CSF (50 ng/ml) for 3 days to generate BMMs, which were subsequently exposed to M-CSF (50 ng/ml) and RANKL (50 ng/ml) for an additional 4 days. OCs were stained for TRAP, and TRAP-positive OCs containing three or more nuclei were counted (10× magnification) (C, top). BMCs derived from WT and hFcγRIIa-Tg mice were plated on calcium phosphate resorbable substrates and cultured in the presence of M-CSF and RANKL for 15 days. The percentage of the resorption area was detected (**C**, bottom). **(D)** RNA was isolated, and Q-PCR was performed for CTSK, MMP9, NFATc1, TRAP and CTR. For Q-PCR, the results are shown as mean ± SD of two pairs of mice and data points represent the mean of three replicates for each mouse. For others, the results are shown as mean ± SD of three pairs of mice.

To elucidate the osteoclast differentiation capabilities of bone marrow cells (BMCs) from hFcγRIIa-Tg and WT mice, we conducted *in vitro* differentiations. Both osteoclast progenitors (OCPs) within BMCs and monocytes/macrophages (BMMs) derived from BMCs possess the potential to differentiate into osteoclasts (25, 26). Non-adherent cells of bone marrow, containing OCPs, directly differentiate into osteoclasts upon treatment with M-CSF and RANKL from the beginning. Notably, as illustrated in **Figure 3A**, the osteoclast differentiation efficiency of hFcγRIIa-Tg mice paralleled that of WT mice. Additionally, the quantity of OCPs exhibited no significant disparity between hFcγRIIa-Tg and WT mice (**Supplementary Figures 4B, C**).

Further investigation involved BMMs, derived from BMCs cultured with M-CSF for 3 days before exposure to RANKL (**Supplementary Figure 4A**). Intriguingly, although there was no difference between the proportions of CD115^+^ CD265^+^ populations of the two mice (**Figure 3B**), BMMs from hFcγRIIa-Tg mice demonstrated a notably more efficient differentiation into osteoclasts *in vitro* compared to those from WT mice (**Figure 3C**), showing both time- and RANKL dose-dependent trends (**Supplementary Figures 2A-D**). Additionally, hFcγRIIa-Tg mice displayed many more characteristic F-actin rings of osteoclasts compared to WT mice (**Supplementary Figure 2E**).

Assessing the functional activity of osteoclasts derived from hFcγRIIa-Tg mice, an *in vitro* pit formation assay was conducted. Notably, osteoclasts from hFcγRIIa-Tg mice exhibited a significant increase in the total pit formation area compared to those from WT mice (**Figure 3C**).

Furthermore, our investigations revealed a substantial upregulation of crucial osteoclastic genes essential for resorptive activity in osteoclasts derived from hFcγRIIa-Tg mice. Notable augmentations were observed in the expression of cathepsin K (CTSK), matrix metalloproteinase 9 (MMP-9), calcitonin receptor (CTR), TRAP, and NFATc1 (**Figure 3D**). These findings strongly suggest that hFcγRIIa expression on BMMs actively promotes osteoclastogenesis *in vitro*.

### 3.4 hFcγRIIa aggravates RANKL-mediated bone loss *in vivo*

**Figure 4:**
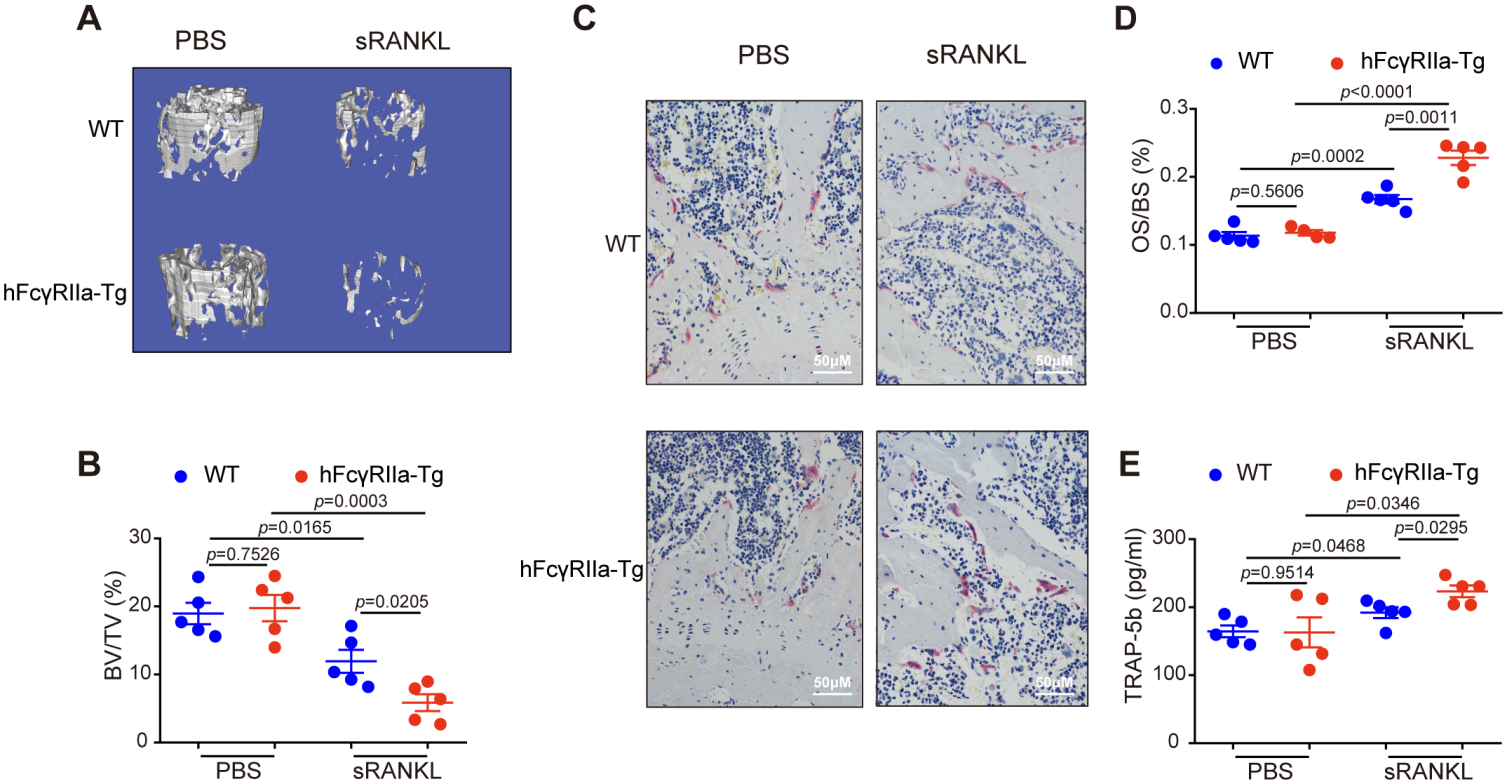
RANKL-induced osteoporosis was more pronounced in hFcγRIIa-Tg mice. WT or hFcγRIIa-Tg mice were injected intraperitoneally with sRANKL (2.0 mg/kg) or an equal volume of PBS at 24 h intervals for 3 days. The mice were sacrificed 1.5 h after the final injection. **(A, B)** The femurs of WT and hFcγRIIa-Tg mice were scanned using μCT. Representative 3D reconstructions of trabecular bone in femurs are shown in **(A)** The BV/TV was analyzed in **(B)**. **(C, D)** Representative images of TRAP-stained femur bone sections (40× magnification) in **(C)** OS/BS are shown in **(D)**. **(E)** TRAP-5b in the serum was determined via ELISA. The results are shown as mean ± SD of five pairs of mice.

Osteoclast (OC) differentiation hinges on signaling pathways activated by the RANKL/RANK interaction (27). Within the joints of inflammatory arthritis patients, RANKL expression by synovial cells and activated T cells is believed to contribute significantly to observed joint destruction in rheumatoid arthritis (6). To investigate hFcγRIIa’s role in RANKL-mediated bone loss *in vivo*, we administered recombinant soluble RANKL (sRANKL) to both WT and hFcγRIIa-Tg mice aged 6 to 8 weeks. Notably, μCT data revealed induced bone loss in both WT and hFcγRIIa-Tg mice, yet the latter exhibited markedly more severe bone loss compared to WT counterparts (**Figures 4A, B**; **Supplementary Figures 3A-C**). Moreover, post sRANKL injection, the number of OCs per bone surface significantly increased in hFcγRIIa-Tg mice compared to their WT littermates, indicating the observed osteopenic phenotype stemmed from an escalated abundance of OCs *in vivo* (**Figures 4C, D**). Additionally, serum levels of TRAP-5b were notably elevated in the hFcγRIIa-Tg mice within the sRANKL group (**Figure 4E**). These findings underscore that, relative to WT mice, hFcγRIIa-Tg mice exhibited significantly heightened OC differentiation and bone resorption within the sRANKL-induced model. This points to the pivotal role of hFcγRIIa in exacerbating RANKL-mediated bone loss *in vivo*.

### 3.5 Mechanistic insights into hFcγRIIa-mediated osteoclast differentiation

**Figure 5:**
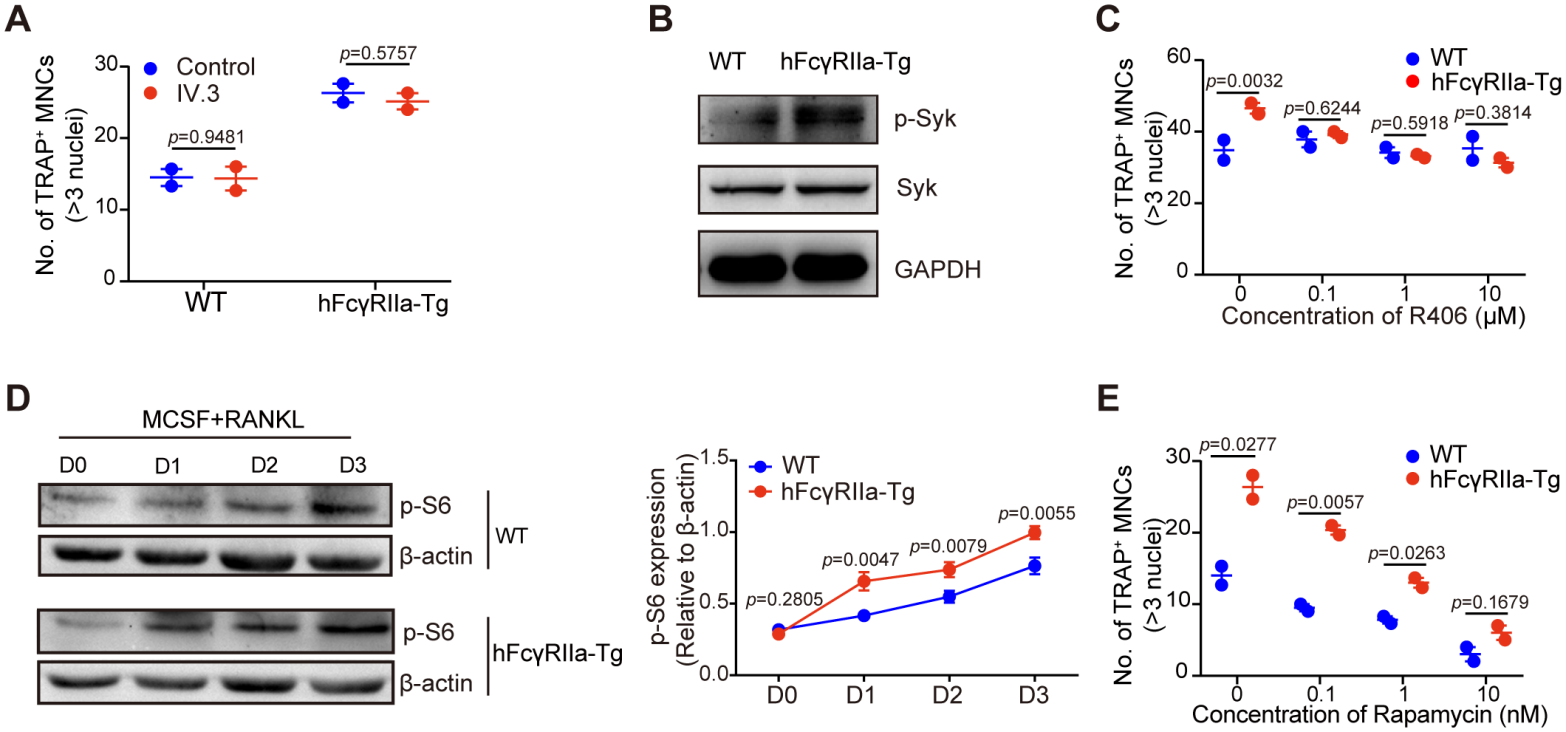
hFcγRIIa promotes RANKL-driven OCs by activating the mTOR-pS6 axis. **(A)** Role of soluble IV.3 in OC differentiation. BMCs were stimulated with or without soluble IV.3 (10 μg/ml) for 3 days in the conventional culture system, followed by TRAP staining and microscopic evaluation. **(B)** Syk phosphorylation in freshly isolated bone marrow cells from mice was measured via WB. **(C)** R406, a Syk kinase inhibitor, was added to the conventional culture system, and the cells were cultured for 3 days before TRAP staining and microscopic evaluation were performed. **(D)** WB detection of p-S6 in cells cultured in the presence of M-CSF and RANKL for 0, 1, 2, or 3 days. **(E)** Rapamycin, an mTOR inhibitor, was added to the conventional culture system, and the cells were cultured for 3 days before TRAP staining and microscopic evaluation. The results are shown as mean ± SD of two pairs of mice and data points represent the mean of three replicates for each mouse.

Investigating the potential ligands for hFcγRIIa, we explored whether fetal calf serum (FCS) containing immune complexes (ICs) might trigger hFcγRIIa activation. Surprisingly, blockade with the specific hFcγRIIa antibody IV.3 demonstrated no significant impact on osteoclast (OC) differentiation *in vitro* (**Figure 5A**). This suggested that hFcγRIIa’s promotion of OC differentiation might not rely on cross-linking with ICs. Our focus shifted to the intracellular signaling cascades behind over-expressed hFcγRIIa’s action. Assessing bone marrow cells from hFcγRIIa-Tg mice revealed spontaneous Syk phosphorylation, absent in WT counterparts (**Figure 5B**). The Syk kinase inhibitor R406 dose-dependently inhibited OC differentiation solely in hFcγRIIa-Tg-derived bone marrow cells, leaving WT cells unaffected (**Figure 5C**). These observations suggested a critical role of Syk activation in hFcγRIIa-mediated OC differentiation. Furthermore, delving deeper into downstream pathways, we found heightened expression of p-70S6K, a key component of the mTOR signaling pathway (28–30), in hFcγRIIa-Tg mice (**Figure 5D**). The mTOR inhibitor rapamycin more efficiently curbed OC differentiation in hFcγRIIa-Tg-derived bone marrow cells compared to their WT counterparts (**Figure 5E**). These findings collectively point to hFcγRIIa’s ability to stimulate OC differentiation through the self-activation of the Syk-mTOR-p70S6 pathway.

### 3.6 Crosslinking of hFcγRIIa inhibits OC differentiation

**Figure 6:**
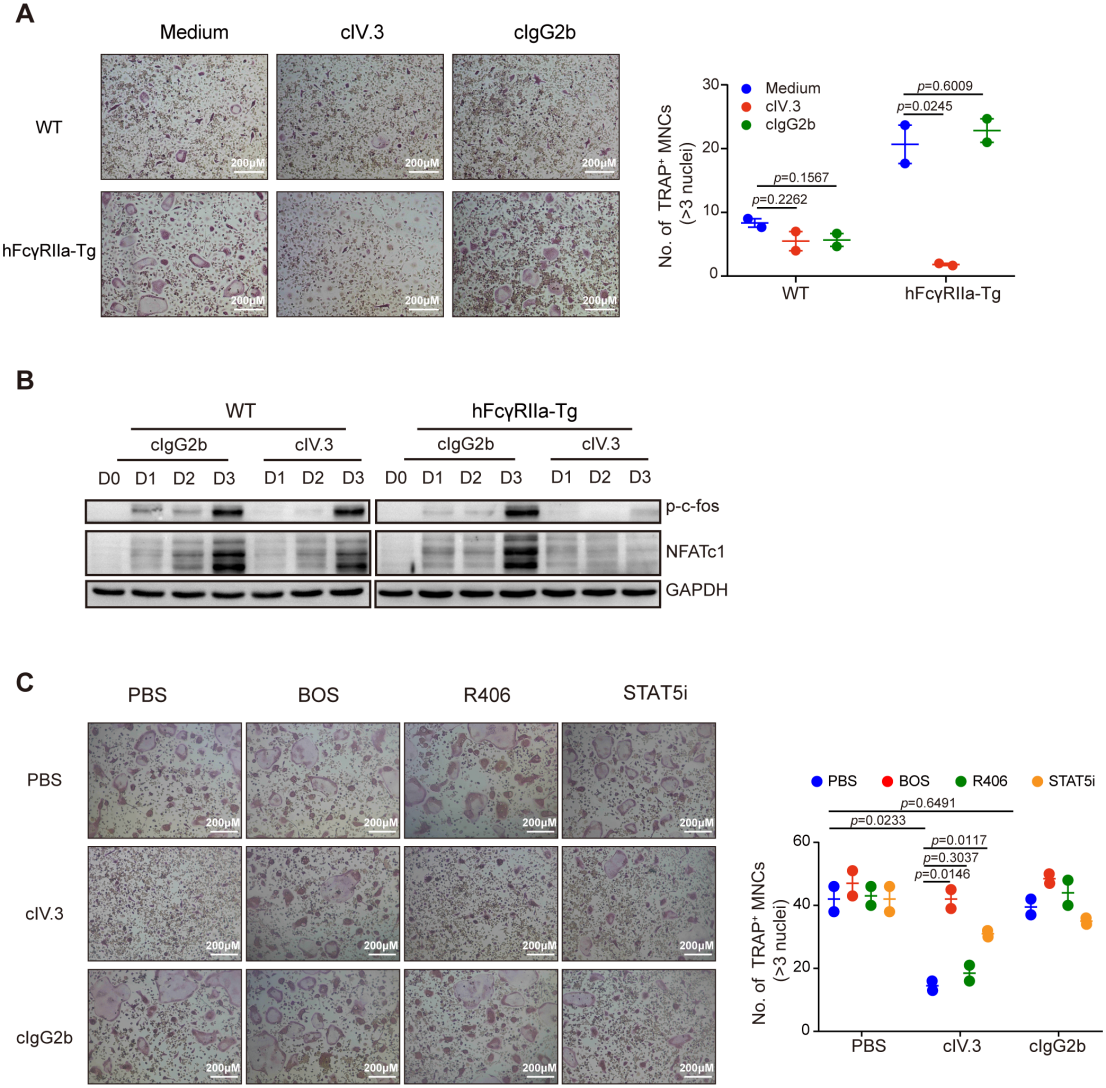
Crosstalkage of hFcγRIIa with cIV.3 blocks osteoclastogenesis **(A)** cIV.3 blocks OC differentiation. BMCs were stimulated with or without cIV.3 (1 μg/ml) for 3 days in the conventional culture system, followed by TRAP staining and microscopic evaluation. **(B)** WB detection of p-c-fos and NFATc1 in cells cultured in the conventional culture system with cIV.3 for 1, 2, and 3 days. **(C)** Treatment with the cIV.3 inhibitor but not R406 reversed the inhibitory effect of cIV.3 cross-linking. BMCs were exposed to cIV.3 (1 μg/ml) + MCSF (50 ng/ml) for 3 days with or without inhibitors, such as R406 (1 nM), bosutinib (100 nM) and a STAT5 inhibitor (50 μM), followed by TRAP staining (40× magnification). The results are shown as mean ± SD of two pairs of mice and data points represent the mean of three replicates for each mouse.

Building upon prior evidence of hFcγRIIa’s contrasting effects on human osteoclast (23), we investigated the impact of hFcγRIIa cross-linking on mouse osteoclast differentiation. Notably, plate-coated IV.3 (cIV.3), a mouse IgG2b isotype specifically binding hFcγRIIa, was employed to cross-link hFcγRIIa. The plate-bound mIgG2b isotype, which show low-affinity to hFcγRIIa (31), had no influence on OC differentiation. Strikingly, hFcγRIIa cross-linking via cIV.3 resulted in a complete reversal of RANKL-induced classical OC differentiation regardless of exposure to cIV.3 for 3 or 6 days (**Figure 6A** and **Supplementary Figures 5A, B**). Meanwhile, cIV.3 could generate small multinuclear cells (SMCs) independent of RANKL (**Supplementary Figures 6A, B**). Concurrently, the expression of crucial transcription factors NFATc1 and c-fos essential for OC differentiation was entirely inhibited upon hFcγRIIa cross-linking (**Figure 6B**). Previous findings regarding the inhibitory role of hFcγRIIa activation on human OC differentiation via STAT5 activation prompted our exploration into similar mechanisms in mice (23). Consistently, a STAT5 inhibitor reversed the inhibitory effect of cIV.3 cross-linking on OC differentiation (**Figure 6C**). Furthermore, the involvement of nonreceptor tyrosine kinase c-Abl, known to activate STAT5 upon hFcγRIIa cross-linking, was evident in our study (23). Interestingly, the Src/Abl inhibitor Bosutinib successfully reversed the inhibition of OC differentiation induced by hFcγRIIa cross-linking, while the Syk inhibitor R406 did not produce the same effect (**Figure 6C**). Similarly, Bosutinib/STAT5 inhibitor suppressed SMCs formation, while R406 did not (**Supplementary Figure 6C**).

These intricate observations delineate the multifaceted role of hFcγRIIa in governing osteoclast differentiation. Low-level activation of hFcγRIIa predominantly promotes osteoclast differentiation through the Syk-mTOR pathway. In contrast, highly cross-linked hFcγRIIa activates the c-Abl-STAT5 pathway, exerting an inhibitory effect on classical osteoclast differentiation.

## 4 Discussion

Previous studies have linked hFcγRIIa to an increased risk of rheumatoid arthritis (RA) and highlighted its role as an activating Fcγ receptor (32–34). Our earlier work unveiled a unique subset of osteoclasts generated by fully cross-linking hFcγRIIa with IgG immune complexes (ICs), independent of RANKL and inflammatory cytokines (23). This study further expands on our prior findings, delineating the dual regulatory role of hFcγRIIa in osteoclastogenesis.

Information provided by Jackson Laboratory indicates that the genomic clone used in the transgene encodes the R131 polymorphic form of hFcγRIIa. Ten copies of the transgene were estimated to have inserted into the genome. Transgenic mice express hFcγRIIa on mouse platelets and macrophages at levels equivalent to that observed in human cells (35). Previous studies have indeed shown that a spontaneous, multisystem autoimmune disease developed in aging (>20 weeks) hFcγRIIa transgenic mice, and most had antihistone antibodies and antinuclear antibodies (ANAs) above background levels with elevated levels of TNF-α (22, 36). Our investigation initially revealed ligand-independent Syk phosphorylation in bone marrow cells of hFcγRIIa transgenic mice. Unlike other FcγRs, hFcγRIIa has both IgG binding sites and signal-transducing ITAMs in the same polypeptide chain. Early studies have indicated that hFcγRIIa exists as a noncovalent dimer, even in the absence of IgG ligand. This is also consistent with previous x-ray crystallographic studies (37–39). that may be responsible for causing Syk phosphorylation. Here, we first identified ligand-independent Syk phosphorylation of hFcγRIIa in transgenic mice, which may also help to explain the spontaneous autoimmune disease observed in Tg mice.

The phosphorylated ITAMs of FcγRs are known to be important costimulatory signals that promote RANKL-driven OCs by primarily activating the Syk-PLCγ-Ca^2+^ pathway and ultimately enhancing the induction of NFATc1, an indispensable factor in RANKL-driven OCs (27, 40). Unfortunately, Ca^2+^ is essential for osteoclastogenesis (41), and its chelator (BAPTA-AM) significantly inhibits OCs in both transgenic and wild-type mice *in vitro*, making it challenging to evaluate the role of hFcγRIIa-mediated Ca^2+^ flux to OCs (data not shown). mTOR, a downstream molecule of Syk, plays dual roles in promoting and inhibiting OC differentiation and is influenced by cell differentiation stage. Here, our data support the positive role of mTOR, particularly the downstream molecule S6, in osteoclasts, which promotes osteoclastogenesis and bone resorption by inhibiting autophagy (28, 42, 43). Thus, our results revealed that the Syk-mTOR-pS6 axis was a critical component of the hFcγRIIa-induced promotion of osteoclastogenesis. Furthermore, as previously reported in the literatures, chronic inflammatory diseases such as rheumatoid arthritis, ankylosing spondylitis, and inflammatory bowel disease are frequently accompanied by osteoporosis (44, 45). Proinflammatory cytokines directly or indirectly regulate osteoclastogenesis and bone resorption providing a link between inflammation and osteoporosis. TNF-α, IL-1, IL-6, and IL-17 are the most important proinflammatory cytokines triggering inflammatory bone loss (46–48). It has also been suggested that inflammatory bone loss is a result of the hyperactivity of osteoclasts and the hypoactivity of osteoblasts, which leads to a profound net reduction in bone mass (49). Based on these, the overall increased inflammation in hFcγRIIa mice may also be partially responsible for bone loss *in vivo*. However, we have not denied the existence of certain other ligands, e.g., CRP, in addition to proinflammatory cytokines and numerous chemokines, due to the complexity of the microenvironment, which could also potentially play an influence in promotion of osteoclastogenesis *in vivo* (50).

Soluble IV.3, an hFcγRIIa-blocking antibody, is considered to lack the intrinsic capacity to trigger signal transduction. However, *in vitro* studies have shown that IV.3 can activate hFcγRIIa when extensively aggregated with goat anti-mouse IgG (51). Our previous research has also demonstrated that cIV.3, which simulates the deposition of IgG ICs, could cross-link high-avidity hFcγRIIa to human monocytes to activate intracellular signaling *in vitro* (23). Due to the potential interaction of IV.3’s Fc fragment with other murine FcRs (18) and the challenges in preparing IV.3-Fab (51, 52), we opted to utilize an isotype control antibody (mouse IgG2b). However, the mouse IgG2b-treated group did not exhibit the reported promotion of OCs in wild-type mice (9) possibly due to the low coating amount of IV.3 in our study.

In some previous studies showing RANKL-potentiating effect of ICs, monocytes were given IgG IC at a later time point during incubation with RANKL and M-CSF (9, 14). However, our results showed that cross-linking of hFcγRIIa by cIV.3 completely reversed RANKL-induced classical osteoclastogenesis regardless of exposure to cIV.3 for 3 or 6 days. Notably, it is difficult to set more time points in our experimental system since osteoclastogenesis in mice takes only 2~3 days after RANKL addition, which is much less than the time of human OC differentiation (~12 days). In addition, our previous experiments on human OC differentiation did not observe significant IC-mediated potentiation of RANKL-induced OC formation irrespective of the time points of IC exposure (23). This inconsistency with the literature may be due to differences in the details of the experimental setup and the acylation state of the ICs (14).

Despite Syk being well known as the major kinase in the hFcγRIIa signaling pathway, other Syk-independent signaling pathways (53, 54) such as the c-Abl pathway, also exist (55, 56). The c-Abl-JAK-STAT5 signaling pathway is also involved in OC differentiation (57, 58). According to emerging research, the STAT signaling pathway plays an important role in bone development and metabolism (59). Recently, STAT5 was shown to negatively regulate the bone resorption of osteoclasts by inducing Dusp gene expression (60). Consistent with these findings, osteoclastogenesis was entirely blocked by activating the critical c-Abl-STAT5 axis in the presence of hFcγRIIa-specific cross-linking IV.3. Furthermore, inhibiting the c-Abl-STAT5 axis was sufficient to rescue osteoclastogenesis in the presence of RANKL. Our results provide further evidence that the c-Abl-STAT5 pathway is one of the critical pathways for suppressing OC differentiation.

In the present work, we were unable to investigate the impact of IV.3 on mouse OCs and bone metabolism *in vivo*. According to previous reports, injecting IV.3 into mice can cause serious side effects, such as hypothermia, thrombocytopenia, and type II hypersensitivity reactions (51, 61). However, similar to injection of effector-deficient IV.3 cells, injection of an anti-hFcγRIIa F(ab′)2 fragment (AT-10 F(ab′)2) significantly inhibited arthritis development in hFcγRIIa-Tg mice by shifting hFcγRIIa from the ITAM to the inhibitory ITAM (ITAMi) signaling pathway (34). Indeed, the difference in phosphorylation status of the three tyrosine residues on hFcγRIIa is the intrinsic molecular mechanism of its activation and includes mono-phosphorylation (ITAMi) and dephosphorylation (ITAMa) (62, 63). Therefore, we hypothesized that cross-linking hFcγRIIa might inhibit OC differentiation and reduce bone loss *in vivo*. Notably, our results do not support the hypothesis that ICs may promote OC differentiation *in vivo* (11, 16), as this is a complex result of ICs acting with various FcRs and/or cross-talk with other receptors, such as Toll-like receptors (TLRs).

In conclusion, we demonstrated that hFcγRIIa plays a dual role in osteoclastogenesis and bone homeostasis in mice. Under physiological conditions, hFcγRIIa activates the mTOR-pS6 axis to promote RANKL-driven OC and bone resorption via ligand-independent Syk phosphorylation. However, by specifically crosslinking hFcγRIIa with cIV.3, RANKL-driven OCs through the c-Abl-STAT5 axis was blocked to suppress p-c-fos and NFATc1. Our findings will provide a new theoretical perspective on hFcγRIIa as a potential therapeutic target for treating RA.

## Data Availability

The original contributions presented in the study are included in the article/**Supplementary Material**. further inquiries can be directed to the corresponding authors.
