# Supplementary material for: hFcγRIIa: a double-edged sword in osteoclastogenesis and bone balance in transgenic mice
Source: Front Immunol. 2024 Aug 30;15:1425670. doi: 10.3389/fimmu.2024.1425670 (PMC11392756; doi:10.3389/fimmu.2024.1425670)
Supplement: Supplementary file 1 [file DataSheet1.pdf]

Supplementary Material

Supplementary Material

1 Supplementary Figures and Tables

1.1 Supplementary Figures

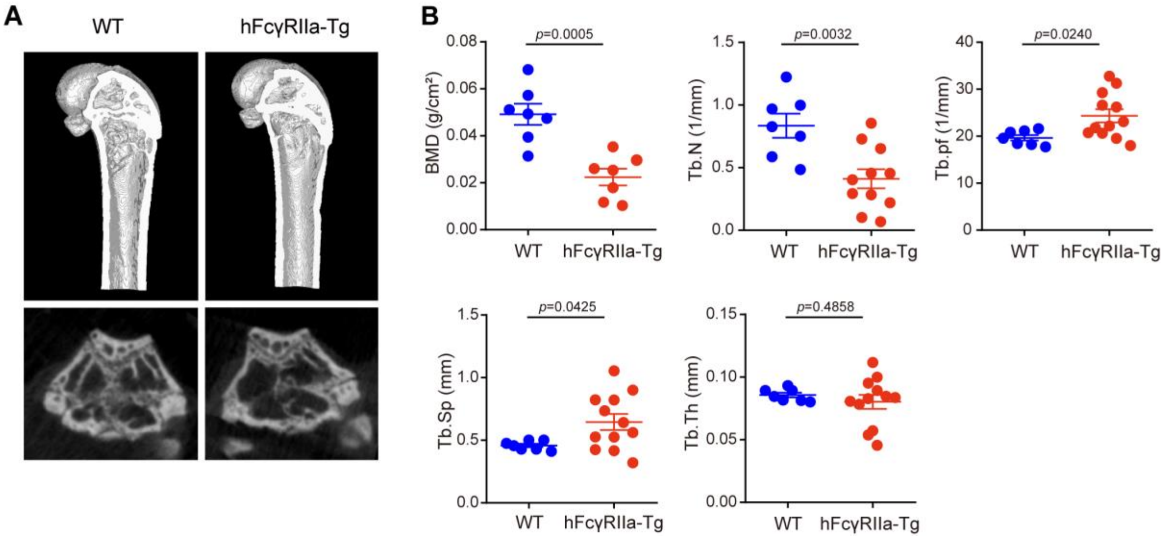

Supplementary Figure 1. hFcγRIIa enhanced bone loss in aging mice (40 weeks). **(A)** Representative μCT images of the femur bone in aging Tg and WT mice. **(B)** Comparative analysis of the bone structural parameters in both mice, including bone mass density (BMD), trabecular number (Tb.N), trabecular bone pattern factor (Tb.pf), trabecular separation (Tb.Sp) and trabecular thickness (Tb.Th). The results are shown as mean ± SD of at least six pairs of mice.

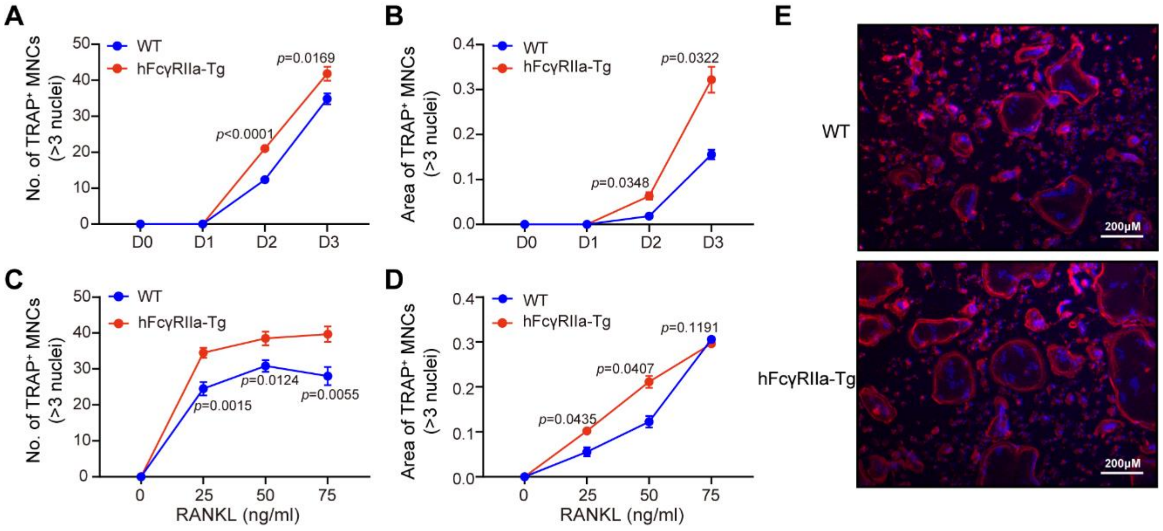

Supplementary Figure 2. hFcγRIIa promotes RANKL-driven OC differentiation in vitro. **(A, B)** Tg mice differentiated into OCs in vitro more efficiently than WT mice in a time-dependent manner. The number of cells with ≥3 nuclei per field. (left), and the area of TRAP-positive cells (right). **(C, D)** Tg mice differentiated into OCs in vitro more efficiently than WT mice in a RANKL dose-dependent manner. The number of cells with ≥3 nuclei per field (left), and the area of TRAP-positive cells (right). **(E)** The typical F-actin rings of OCs. F-actin rings were stained with TRITC-phalloidin (red), and nuclei were counterstained with DAPI (blue). The results are shown as mean ±SD of two pairs of mice and data points represent the mean of three replicates for each mouse.

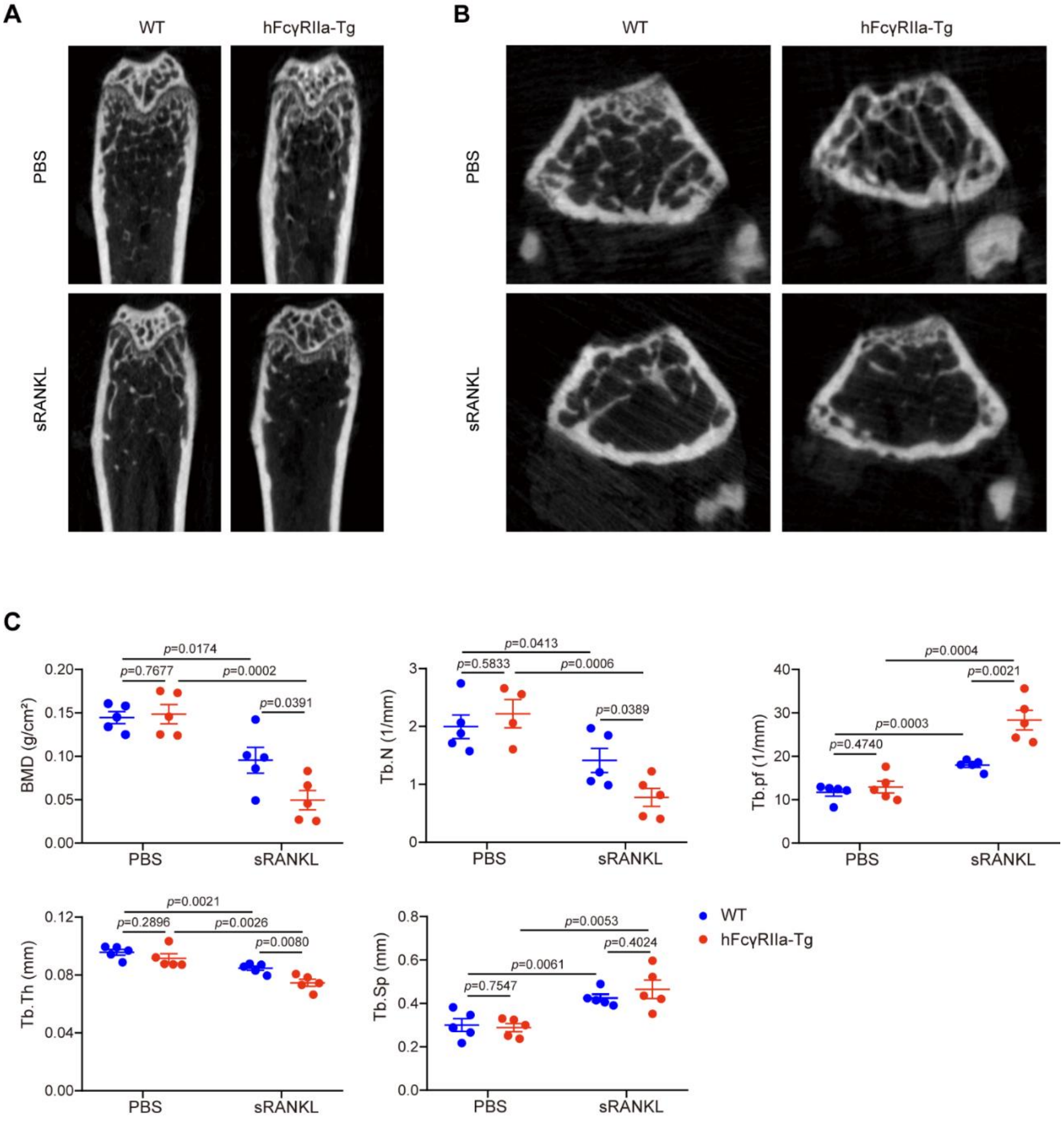

Supplementary Figure 3. Enhanced bone loss induced by RANKL in Tg mice. **(A, B)** Representative μCT images of the femurs in PBS and sRANKL group mice. **(C)** Comparative analysis of the bone structural parameters such as bone mass density (BMD), trabecular number. (Tb.N), trabecular bone

pattern factor (Tb.pf), trabecular thickness (Tb.Th), trabecular separation (Tb.Sp). The results are shown as mean ± SD of five pairs of mice.

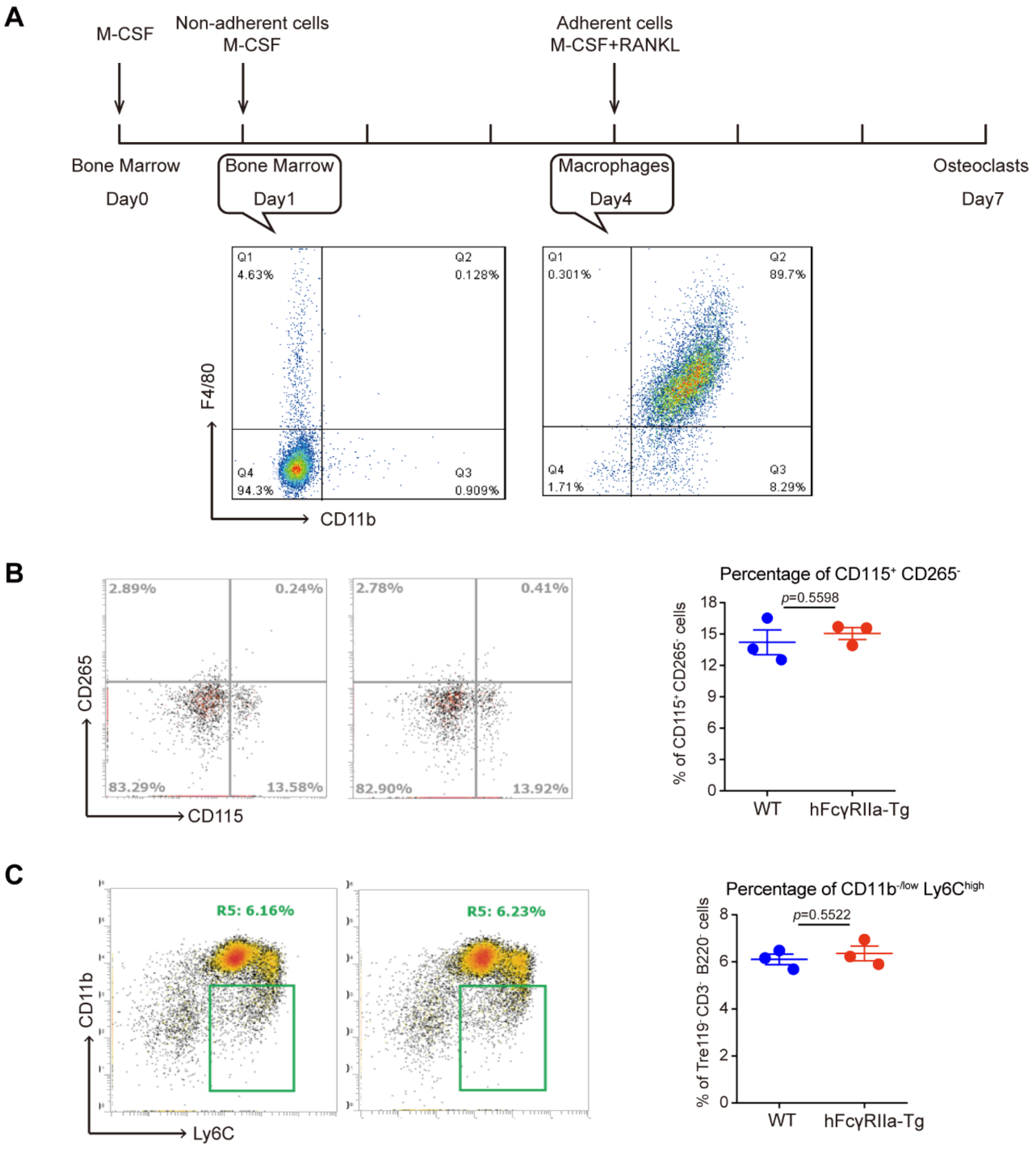

Supplementary Figure 4. OC differentiation strategies and OCPs analysis. **(A)** A detailed step-illustrations of the conventional osteoclast differentiation strategy. **(B, C)** Two classes of OCPs in BMCs, with the cell-surface phenotypes CD115^{+} CD265^{-} and CD11b$^{-/low}$ Ly6C^{high}, did not differ in proportion between WT and Tg mice. The results are shown as mean ± SD of three pairs of mice.

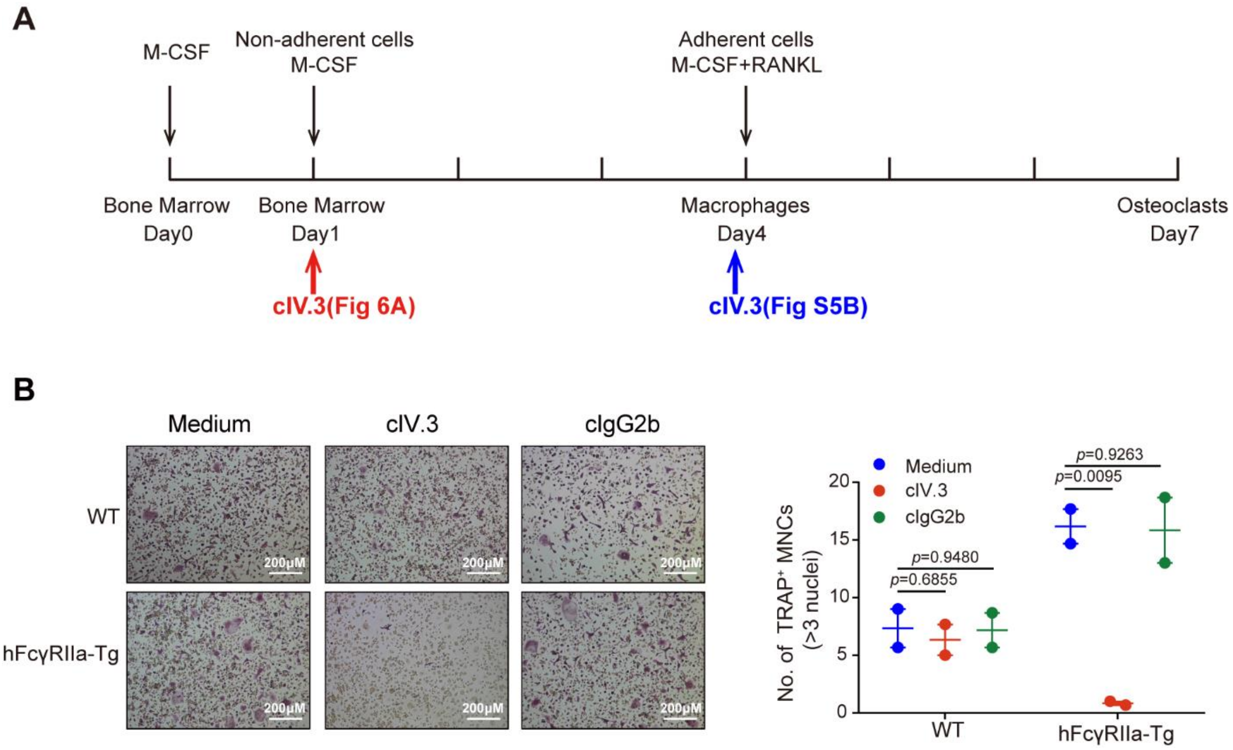

Supplementary Figure 5. Cross-linking hFcγRIIa blocks OC differentiation. **(A)** A detailed step-illustrations of the effect of cIV.3 on osteoclast differentiation. **(B)** cIV.3 blocks OC differentiation even exposed to cIV.3 for 3 days before the TRAP staining. The results are shown as mean ± SD of two pairs of mice and data points represent the mean of three replicates for each mouse.

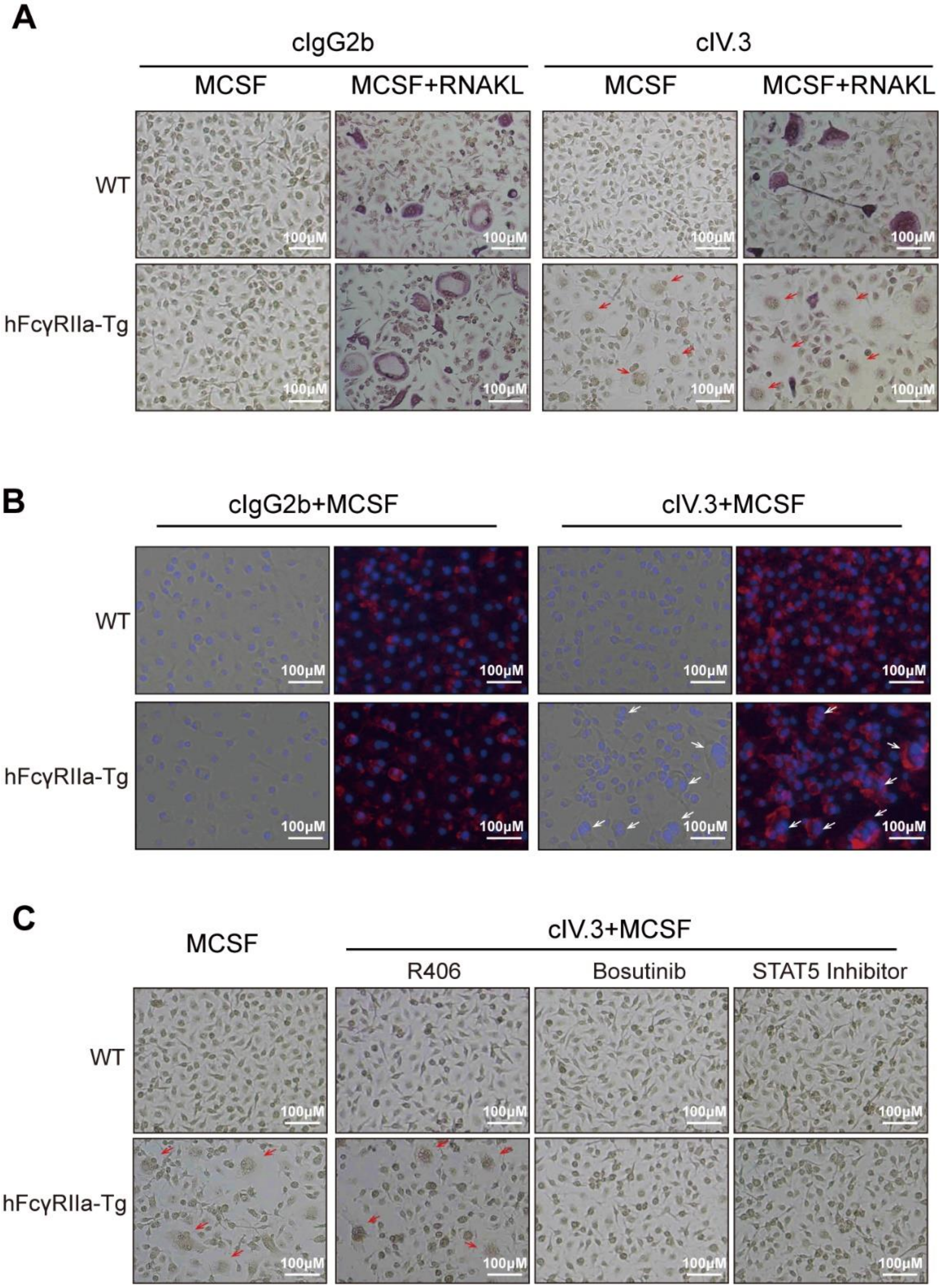

Supplementary Figure 6. Cross-linking hFcγRIIa induces a kind of TRAP-negative multinucleated cells and reversing osteoclastogenesis experiment. **(A, B)** IV.3 but not isotype control antibody (mouse IgG2b) could drive the differentiation of TRAP-negative SMCs independent of RANKL. As shown by the arrow **(B)**, F-actin rings were stained with TRITC-phalloidin (red), and nuclei were counterstained with DAPI (blue). **(C)** Src/Abl inhibitor (Bosutinib) and STAT5 inhibitor but not R406 inhibits the generation of SMCs. The results are shown as mean ±SD of two pairs of mice and data points represent the mean of three replicates for each mouse.

1.2 Supplementary Tables

Gene	Forward primer	Reverse primer
GAPDH	CCTTCCGTGTTCCTACCCC	GCCCAAGATGCCCTTCAGT
CTSK	ATGTGGGTGTTCAAGTTTC	TCAATGCCTCCGTTCT
MMP9	TCCAGTTTGGTGTCGC	GTCCACTCGGGTAGGG
NFATc1	TGGGAGATGGAAGCAAAGAC	TGGGAGATGGAAGCAAAGAC
TRAP	AGACCCAATGCCACCC	GGACCTCCAAGTTCTTATC
CTR	CCTCTTGCCCTTGGGTGCTATC	CTGGGGAGTAAAGAGGGGTATGG

Supplementary Table 1. Primer sequences used for real-time quantitative PCR.
